# Autotuning of Exascale Applications With Anomalies Detection

**DOI:** 10.3389/fdata.2021.657218

**Published:** 2021-11-26

**Authors:** Dragi Kimovski, Roland Mathá, Gabriel Iuhasz, Fabrizio Marozzo, Dana Petcu, Radu Prodan

**Affiliations:** ^1^ Institute for Information Technology, University of Klagenfurt, Klagenfurt, Austria; ^2^ Department of Computer Science, West University of Timisoara, Timisoara, Romania; ^3^ Institute e-Austria Timisoara, Timisoara, Romania; ^4^ DIMES Department, University of Calabria, Rende, Italy

**Keywords:** exascale computing, autotuning, events and anomalies detection, multi-objective optimization, IoT applications

## Abstract

The execution of complex distributed applications in exascale systems faces many challenges, as it involves empirical evaluation of countless code variations and application runtime parameters over a heterogeneous set of resources. To mitigate these challenges, the research field of autotuning has gained momentum. The autotuning automates identifying the most desirable application implementation in terms of code variations and runtime parameters. However, the complexity and size of the exascale systems make the autotuning process very difficult, especially considering the number of parameter variations that have to be identified. Therefore, we introduce a novel approach for autotuning exascale applications based on a genetic multi-objective optimization algorithm integrated within the ASPIDE exascale computing framework. The approach considers multi-dimensional search space with support for pluggable objective functions, including execution time and energy requirements. Furthermore, the autotuner employs a machine learning-based event detection approach to detect events and anomalies during application execution, such as hardware failures or communication bottlenecks.

## 1 Introduction

In the era of the Internet of Things (IoT), massive amounts of data are created continuously, which have to be transferred, processed, and analyzed within tight deadlines. To meet the strict constraints, the emerging exascale computing systems, encompassing high-performance computing systems and large-scale distributed cloud data centers, are utilized (De Maio and Kimovski, 2020). However, the complexity of the exascale systems makes designing and deploying distributed applications a very complex task, which requires a deep understanding of the exascale system and proper characterization of the applications in terms of runtime parameters (Silvano et al., 2016).

The process of application execution in exascale systems faces many challenges, as it involves the control of millions of threads running on thousands of heterogeneous devices (Balaprakash et al., 2018). The applications, which utilize these systems, need to avoid synchronization, reduce the communication events, and implement complex strategies to avoid failures. To mitigate these challenges, the research field, known as autotuning, has gained attention (Jordan et al., 2012). In general, autotuning refers to the process of automatic identification of the most desirable application implementation in terms of code variations and runtime parameters. Autotuners can produce efficient code versions and runtime parameters of complex applications by creating multiple code variants that are empirically evaluated on the target systems. Furthermore, they can identify code configurations, which might not be intuitive for the application developers and therefore lead to performance improvements.

However, the complexity and size of the exascale systems make the autotuning process for exascale applications very difficult, especially considering the number of parameter variations that have to be identified (Durillo and Fahringer, 2014). This problem is further aggravated by the high probability of failures in systems containing thousands of nodes. To address the problem of autotuning, the concurrent approaches are primarily focused on improving the execution time of the application and reducing energy consumption during execution. However, these approaches are not suitable for the exascale systems, as they do not consider the high heterogeneity of the exascale environments. Furthermore, they can not detect events and anomalies during application execution, such as possible hardware failures or communication bottlenecks, which can be used to mitigate application failures and further improve execution efficiency.

Therefore, in this article, we introduce the ASPIDE^1^ autotuning and optimization algorithm that considers multi-dimensional search space with pluggable objectives, including execution time and memory utilization. Moreover, to further improve the application execution, the ASPIDE approach utilizes a machine learning (ML) based events detection approach, capable of identifying point and contextual anomalies (Zhang, 2007). In general, the ASPIDE autotuner assists the developers in understanding the non-functional properties of their applications by making it easy to analyze and experiment with the input parameters. The autotuner further supports them in exposing their obtained insights using tunable parameters. Lastly, we evaluated the ASPIDE autotuning approach in a real execution environment that considers a representative exascale application focusing on social media platforms.

Therefore, the contributions of this paper are as follows:

1. Background review of the autotuning practices for distributed applications in the converging high-performance systems and cloud data centers
2. Multi-objective autotuning approach with pluggable objectives for identifying optimal application execution parameters
3. Machine learning-based anomalies and event detection engine, capable of detecting anomalies during application execution, including hardware failures and communication bottlenecks, thus constraining the multi-objective autotuning approach

The remainder of the paper is structured as follows. Section 2 discusses related work. Section 3 describes the proposed ASPIDE autotuning model with pluggable objective functions. Section 4 presents the novel ASPIDE events and anomalies detection engine. Section 5 describes the ASPIDE platform and the interactions between the autotuning and anomalies detection engine. Section 6 describes a social media case study application, which has been evaluated using a real testbed in Section 7. Section 8 concludes the article.

## 2 Related Work

Autotuning can be applied to the library, code, or application level. In this work, we focus our efforts on application-level autotuning, as many exascale applications have been developed in such a manner that permits the expression of tunable parameters for various problem sizes or code variants. The approaches for application-level tuning can be divided into two categories, based on the optimization objectives: single objective and multi-objective.

### 2.1 Single-Objective Approaches

Tiwari et al. (2009) explored a parameter tuning approach based on a Parallel Rank Ordering (PRO) algorithm, specifically modified to enable offline optimization. The PRO algorithm creates an N-dimensional search space that can be explored with an unknown objective function, where N is the number of tunable parameters.

Furthermore, Ren et al. (2008) presented a framework for automatically tuning distributed and parallel applications to hardware systems with software-managed memory hierarchies. The approach utilizes a pyramid search algorithm, specifically tailored for parameter tuning, capable of intelligently choosing the initial search point and using a non-square grid.

Besides, Choi et al. (2010) developed a model for guiding the autotuning process focused on multi-threaded vector processors, such as graphical processing units. However, due to its application domain, the model only optimizes the execution time for sparse matrix-vector multiplication.

Likewise, Muralidharan et al. (2014) provided a library for expressing code variants, together with meta-information on the application, that improves the efficiency of autotuning. The library applies machine learning algorithms to build a model by training with the provided meta-information. When new input is provided, the library can choose appropriate code variants and parameters.

Furthermore, Ansel et al. (2009) introduced a new implicitly parallel language and compiler that enable multiple algorithms to be described for solving a single problem. This allows the autotuner to tune the application at a finer granularity, including data distribution and algorithmic parameters.

From an energy efficiency perspective, Tiwari et al. (2011) extended over the current software tools for autotuning of applications with the main goal to minimize the power requirements while maintaining the application performance. The autotuning process is conducted concerning application-level tunable parameters and the scaling of the processor clock frequency. The tunable parameters are identified and later explored via an offline search strategy.

### 2.2 Multi-Objective Approaches

A search-based multi-objective approach has been described by Tapus et al. (2002), which utilizes two criteria (e.g., execution time and efficiency) to adjust the tunable parameters of distributed applications and libraries. Furthermore, Jordan et al. (2012) extended this approach by considering both the execution time and compilation time as conflicting objectives.

Moreover, Balaprakash et al. (2013) provided a multi-objective optimization model for autotuning a broad set of applications and architectural designs in high-performance computing. The work explored multiple conflicting objectives, such as time, power, and energy, to provide rich insight intp how to tune the application parameters.

Furthermore, Gschwandtner et al. (2014) presented a multi-objective model for autotuning parallel applications based on trade-off analysis for application execution time, energy requirements, and resource utilization. The model can be applied for high-level parallel programming languages and utilizes a novel method, called RS-GDE3, to tune parallel codes during compilation.

Besides, Ansel et al. (2014) described the OpenTuner framework, which enables the formulation of the code optimization problem as a trade-off. The framework enables the developers to identify potential trade-offs among multiple objectives and provides a significantly richer knowledge of the applications through an application behavior analysis.

### 2.3 Research Gap

The presented research works primarily improve the application’s execution time and the system’s energy consumption from a resource provider perspective. They usually omit or consider the requirements of the users of the applications with lower priority. Besides, they do not consider the memory footprint of the applications, which makes them not applicable for memory-intensive applications that frequently require in-memory processing.

Furthermore, none of them provides means for detecting events and anomalies during execution, such as hardware failures, which can further improve the application execution. Concretely, they only analyze the influence of the parameters on the application execution without considering the identified execution anomalies, which can significantly hinder the autotuning process.

Therefore, our approach improves over these methods by applying a multi-objective approach with pluggable objectives that consider multi-dimensional search space, including memory utilization, execution time, and energy efficiency. Moreover, to further improve the application process, our approach utilizes an events and anomalies detection engine capable of detecting events and anomalies during application execution.

## 3 Autotuning Model

### 3.1 Autotuning Process

The ASPIDE autotuner iteratively seeks optimal application parameter settings tuned for the given exascale infrastructure. As the number and the definition range of tuning parameters are application-specific, the size of the parameter and objective search spaces varies among different applications. Accordingly, with the increasing search space size also the search complexity increases. To speed up and guide the search process on higher scales, the ASPIDE autotuner implements a specifically tailored genetic algorithm. The autotuner is based on a modular design, which allows pluggable objective functions to be used. This means that the main algorithm can take various optimization functions, such as execution time, energy efficiency, or memory footprint and utilization. Therefore, the ASPIDE autotuner can optimize either a single objective or a set of trade-off objectives.

In general, the ASPIDE autotuning approach is based on the well-known NSGA-II algorithm (Kimovski et al., 2016), which moves on each iteration closer towards a Pareto optimal set of application parameters considering anomaly-specific constraints, such as performance anomalies or hardware failures during previous executions. Our approach uses a historical set of previously evaluated application parameter settings to suggest, during each iteration, new application parameter settings. The new application settings are evaluated in a real environment, and the values of the objectives are measured by a Prometheus-based monitoring tool (see Section 5). After the evaluation step is completed, the new application parameter settings are added to the historical setting and shared with the events and anomalies detection engine for the next iteration.

**FIGURE 1:**
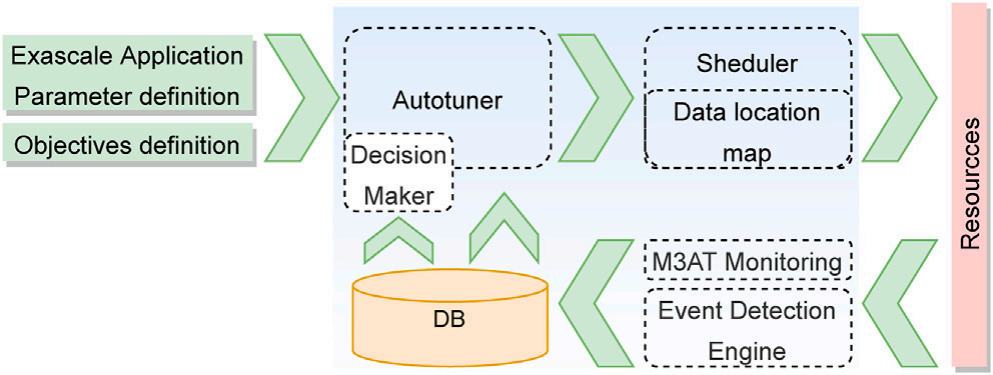
Data flow diagram of the autotuning process.

Figure 1 depicts the data flow diagram of the ASPIDE autotuner. The autotuner receives the structure of the exascale application, together with the defined parameters and pluggable objective functions. Before the autotuning process is started, the ASPIDE autotuner searches for previous execution parameters in a centralized database (DB). The information of the previous executions is used to steer the autotuning process. The output from the autotuner is provided to the application scheduler, which in combination with the data location map, decides on which resources to execute the applications. The execution is continuously monitored by the M3AT monitoring system (see Section 5), and the information is used by the event detection engine (see Section 4) to search for execution anomalies. The information on the execution parameters and the detected anomalies is stored again in the DB. This information can be fetched by the autotuner for further improving the tunable parameters.

### 3.2 Implementation

Algorithm 1 presents the pseudo-code of the ASPIDE autotuner implementation. Note that the pseudo-code shows one iteration of the autotuner. Accordingly, each autotuner iteration can return either a single solution or a set of Pareto solutions concerning the number of objective functions used during optimization.

**FIGURE 2:**
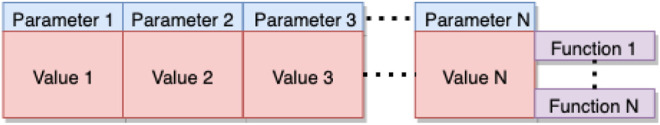
Individual vector for representation of the tunable parameters.

As the first step in each iteration, the population *P* is filled with historical individuals generated and evaluated from previous runs. The individuals depicted in Figure 2 are represented as vectors, where the index of the vector corresponds to a given tunable parameter *S* and the vector field represents a specific parameter value. The individual vector contains additional fields for representing the fitness value of the optimization objectives based on user-defined pluggable objective functions *O*. The utilization of pluggable functions, which the users can define, allows flexible filtering of individuals by adapting the objective definitions *O* and the application parameter definitions *AP* as constraints to match the latest requirements continuously.

Algorithm 1 Multi-objective ASPIDE autotuning algorithm

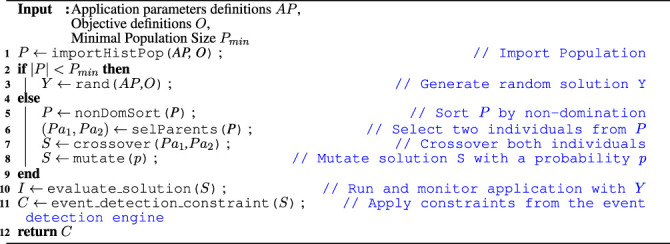

The ASPIDE autotuner relies on an algorithm with a cold start strategy (Lines 2 and 3). This means, if the population size is below a predefined threshold *P*_min_, the ASPIDE autotuner generates individual vectors with random values to create the initial population.

If the population size crosses the minimal threshold, then the autotuner generates new solutions. The solutions are identified based on the fitness value of the objective function, which can be execution time, energy efficiency, or economic costs. This is achieved by the nonDomSort function that sorts the population *P* by non-domination (Line 5). Thereafter, selParents selects two individuals of *P* as parents, and crossover creates a solution *S* out of both selected parents *Pa*_1_ and *Pa*_2_ (Lines 6 and 7). To reduce the risk of identifying only the local minimum, in genetic algorithms, it is common practice to introduce a mutation step (Line 8) that mutates the solution *S* with a probability *p*.

To evaluate the generated application parameters of the solution *S*, i.e., to determine the fitness values, the autotuner defines the function evaluate_solution, which runs and monitors the application returning the new individual *I* (Line 10). The evaluate_solution function is user-defined, which allows for pluggable optimization objectives to be used. The function uses monitoring data from previous runs to determine the fitness values. In the cases where the specific application parameters are evaluated for the first time, only the fitness values from the current execution are considered. Otherwise, the mean value from all previous executions is considered for evaluating the fitness of the given solution (individual).

Subsequently, the algorithm checks with the events and anomalies detection engine (see Section 4) if the evaluated solution satisfies the defined runtime constraints (Line 11). If the anomalies detection engine identified that the evaluated parameters caused performance degradation in the past, such as higher execution time or memory usage, it would discard the solution. Thereafter, the algorithm will start from the beginning to search for other possible parameter values.

Finally, if the solution meets the constraints and the evaluation is completed, the ASPIDE autotuner returns the new individual *I* that will be stored in the monitoring database and considered on the next iteration (Line 12).

## 4 Event Detection Model

### 4.1 Events and Anomalies Definition

In the following section, we define the relationship between the terms events and anomalies. The anomalies are defined as rare events or observations, which are significantly different from most data. Therefore, the anomalies pose an additional level of complexity by their sparse nature, as some anomalies might have an occurrence rate well under 0.01%. Events and anomaly detection can be split up into several categories based on the methods and the characteristics of the available data. The most simple form of anomalies is point anomalies, characterized by only one metric (feature). These types of anomalies are fairly easy to detect by applying simple rules (i.e., CPU is above 70%). Other types of anomalies are more complex but ultimately yield a much deeper understanding of the inner workings of a monitored exascale system or application. These types of anomalies are fairly common in complex systems.

Contextual anomalies are extremely interesting in the case of complex systems. These types of anomalies happen when a certain constellation of feature values is encountered. In isolation, these values are not anomalous, but when viewed in context, they represent an anomaly. These types of anomalies represent application bottlenecks, imminent hardware failure, or software misconfiguration. The last major type of anomalies, which is relevant, is temporal or sometimes sequential anomalies where a certain event takes place out of order or at the incorrect time. These anomalies are significant in systems with a strong spatiotemporal relationship between features, which is very much the case for exascale metrics.

### 4.2 Events and Anomalies Detection Engine Architecture

**FIGURE 3:**
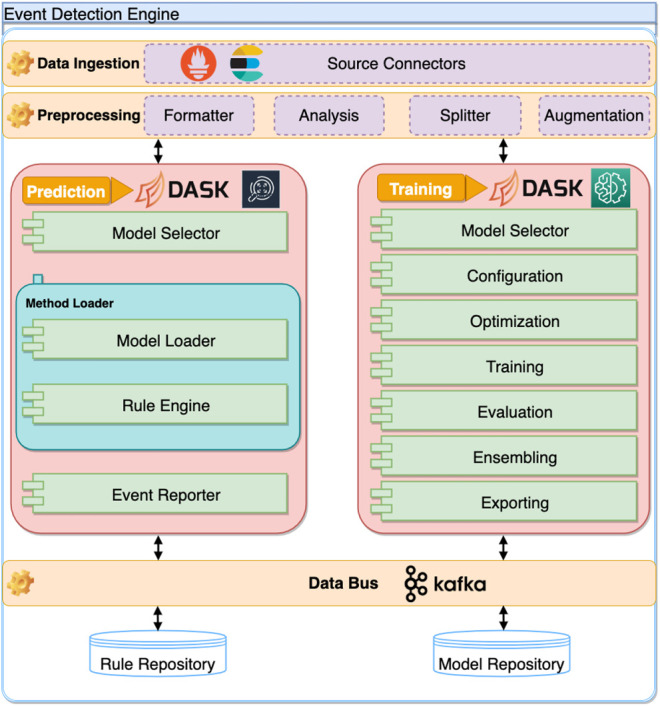
The architectural model of the ASPIDE event detection engine.

The Events and Anomalies Detection Engine Architecture (EDE) contains a set of main components, which are based on lambda type architecture and divided into three layers: data ingestion, data preprocessing, and training and prediction, depicted in Figure 3. First, due to the heterogeneous nature of most modern computing systems (including exascale and mesh networks) and the substantial variety of solutions that could constitute monitoring services, the data ingestion component has to be able to fetch data from a plethora of monitoring systems using various data formats. The data ingestion component implements a connector sub-module that serves as adapters for connecting different monitoring services. Furthermore, this component can also load data directly from static files in various formats, including HDF5, CSV, JSON, or raw format. The ASPIDE EDE supports data ingestion directly via query from the monitoring solution or streamed queuing service to aid the event detection methods further. This reduces the time between the event that was observed and detected.

Afterward, the preprocessing component gathers the raw data from the data ingestion component and applies the required transformations. It handles data formatting (i.e., one-hot encoding), analysis (i.e., statistical information), splitter (i.e., splitting the data into training and validation sets), and finally augmentation [i.e., oversampling and undersampling (He et al., 2008)]. This component is also responsible for any feature engineering of the incoming monitoring data.

The training and prediction component is divided into two sub-components. The training sub-component is used to instantiate and train machine learning models that can be used for event detection. The end-user can configure the hyperparameters of the selected machine learning models and run automatic optimization on these (i.e., Random Search and Bayesian search). Users cannot only set the parameters to be optimized but also define the objectives of the optimization. More specifically, users can define what should be optimized, including but not limited to the predictive performance of the machine learning model. The training sub-component handles the evaluation of the created predictive model on a holdout set. Current research and rankings of machine learning competitions show that creating an ensemble of different methods may yield statistically better results than single model predictions (Dietterich, 2000). Because of this, we included ensembling to the training sub-component. The trained and validated models have to be saved in such a way to enable easy instantiation in a production environment.

Once a predictive model is trained and validated, it is saved inside a model repository. Each saved model has to have metadata attached to it denoting its performance on the holdout set and other relevant information such as size and throughput.

Lastly, the prediction sub-component encompasses retrieving the trained model from the model repository and feeding metrics from the monitored system. The prediction sub-component can either use the trained models to perform inference, detect events and anomalies, or utilize a rule-based approach. In the case a rule-based approach is used, the prediction component has to include a rule-based engine and a rule repository. Naturally, detection of anomalies or any other events is of little practical significance if there is no way of handling them. Therefore, we include a component that tries to resolve the underlying issues once the event has been identified. When an event or anomaly is detected, the ASPIDE EDE is responsible for storing the information within the monitoring system and signaling this to both the ASPIDE autotuner and the scheduler. The information from the prediction component is used by the autotuner and the scheduler in the ASPIDE system to constrain the application execution parameters and resources, which may induce anomalies, such as communication bottlenecks or system failures. All anomalies are currently exported via the EDE data bus, which is implemented based on Kafka topics (Garg, 2013).

### 4.3 Anomalies Induction and Detection Methods

The ASPIDE EDE uses an anomaly induction tool capable of inducing anomalies both on a single node or distributed systems spawning across several nodes. This allows us to validate any results we might obtain fully.

The anomaly induction tool is designed to allow users to define different anomaly induction sessions. Users can select from a few predefined anomalies and create custom distributions that will be executed. This results in logs which, when combined with metrics collected using a monitoring solution, result in labeled data sets.

Related to the anomalies detection, we primarily utilize RandomForest (Svetnik et al., 2003), which is an ensemble learning method that constructs multiple decision trees. The RandomForest algorithm tends to overfit and provide degraded out-of-sample performance compared to other methods. Therefore, the ASPIDE EDE supports additional ML algorithms, such as the XGBoost (Chen and Guestrin, 2016), which, similar to RandomForest, is a decision tree-based ensemble method. One of the main differences is that it utilizes gradient boosting. In recent years, it has become the preferred ML method for small/medium well-structured tabular data. Lastly, the ASPIDE EDE also supports a Deep Neural Network (DNN) implemented in Tensorflow^2^.

The parameter space available for these methods is quite extensive. Thus, we decided on using a guided hyperparameter optimization (HPO) method, namely, a genetic algorithm-based one implemented using the DEAP framework^3^. We used the scikit-learn wrapper from Tensorfow to enable us to expose training hyperparameters and topological features from the event and anomaly detection models. Thus, in the case of the DNN model, the genetic algorithm can also add or remove layers (maximum of four layers) and set the number of neurons from each of these densely connected layers.

## 5 ASPIDE System Architecture

The multi-objective autotuner and machine learning-based events and anomalies detection engine have been integrated within the ASPIDE exascale framework to exploit massive parallelism (Talia et al., 2019). The ASPIDE framework exposes analyses and associates a vast set of application execution parameters with the main goal to improve the application performance in exascale systems. The key insight behind such an approach is that the source of a bottleneck in data-intensive applications is often not where it is detected (i.e., where the data is processed with a high communication or thrashing overhead) but where it is allocated. The ASPIDE approach enables the efficient extension of the GrPPi unified programming model (del Rio Astorga et al., 2016) with autotuning of high-performance, automatically adaptable, and tunable data-intensive exascale applications. The ASPIDE system enables experts and programmers to build solutions using mechanisms that go beyond contemporary approaches’ capabilities by putting static and dynamic optimization and code generation technologies under their control.

**FIGURE 4:**
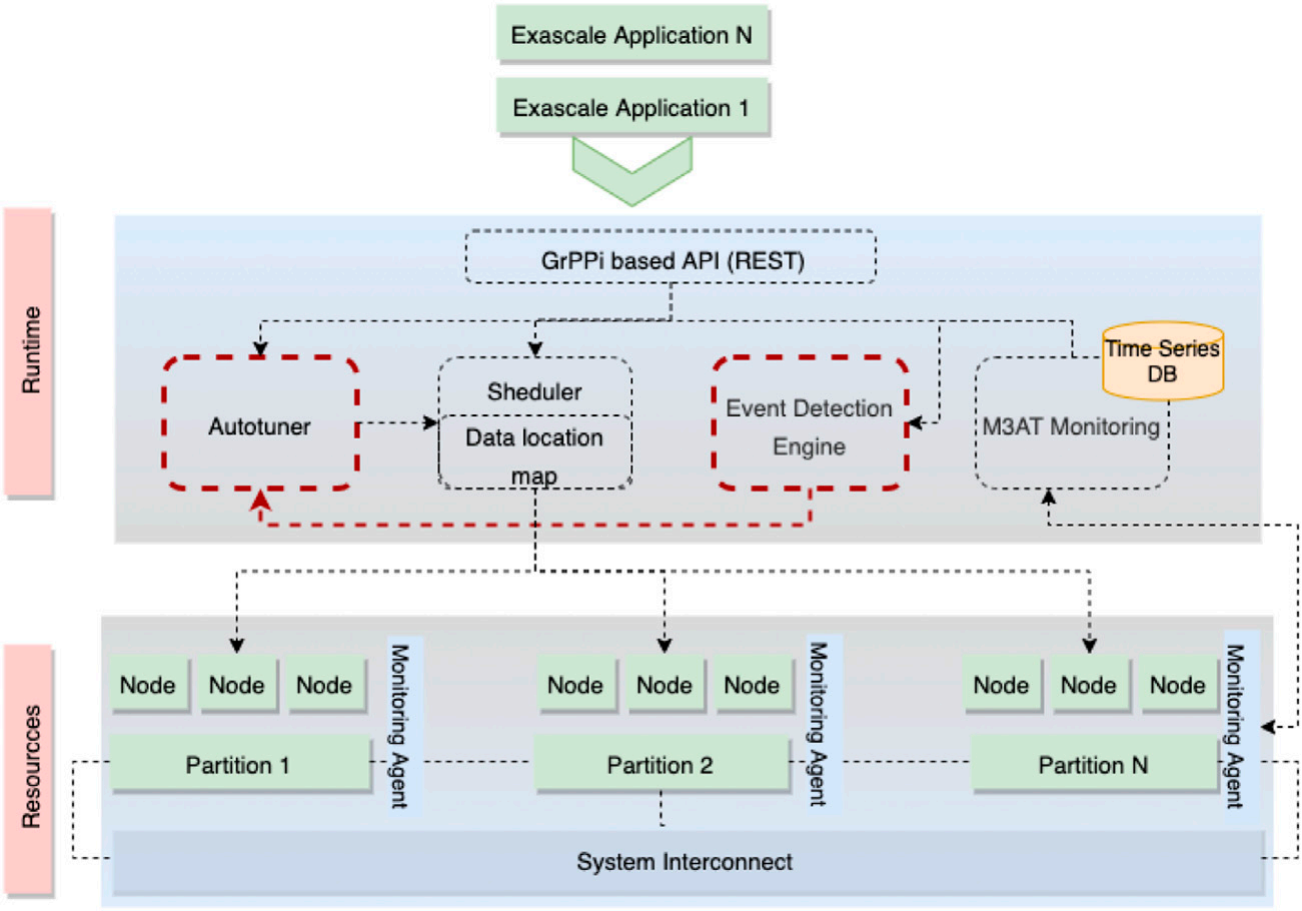
Top view of the ASPIDE system architecture.

The ASPIDE system depicted in Figure 4 consists of the following main components:

• Application Programming Interface (API). The ASPIDE API allows the developers to define their applications transparently. It is based on the GrPPi generic parallel pattern interface for stream processing, which facilitates the development of distributed and parallel applications on exascale systems by concealing the complexity of the concurrency mechanisms.
• Applications scheduler. The ASPIDE scheduler is an inter-changeable service module that delivers pending tasks to the available resources using task metadata and monitoring information for exploiting computing resources and data locality. Therefore, it allows the utilization of different scheduling algorithms. It is capable of supporting three types of queues: sequential, parallel, and not-ready tasks.
• Autotuner with pluggable optimization functions. The ASPIDE autotuner enables developers to utilize pluggable optimization functions for tuning the application runtime parameters. It utilizes Pareto based multi-objective optimization algorithm that iteratively, with each execution, seeks optimal application parameters tuned for given exascale infrastructure.
• Events and Anomalies Detection Engine. The ASPIDE EDE handles the processing of raw monitoring data from the execution of the applications to identify relevant events and anomalies, leading to an application or system failure.
• Scalable monitoring architecture. The ASPIDE monitoring architecture (Kashansky et al., 2020) is based on the Prometheus open-source monitoring system that enables recording numeric time series data. It supports a collection of multi-dimensional data related to the application execution and the hardware system performance.

### 5.1 System Interaction

This section defines the data flow among the components of the ASPIDE system, with the main focus on the interaction between the autotuner and the events and anomalies detection engine. We illustrate the interaction through an usage example. First, the application owners use the ASPIDE API to describe and parallel their application based on the GrPPi language. The ASPIDE API supports an MPI + X model, adapted for distributed memory systems at a large scale. It utilizes a task-based back-end for an exascale environment and employs queues for identifying the relations between the tasks and data. Afterwards, when the application is described, and all dependencies between the components are identified, the application is sent to the autotuner and the scheduler, which accept the application’s tasks in one of the three queues (sequential, parallel, and not-ready). The scheduler can use different scheduling algorithms for the different queues. Afterward, the scheduler sends a request to the autotuner for optimal execution parameters. The request contains a query on which application parameters to be used for the given available infrastructure, such as the number of threads and data size. The autotuner, based on the optimization functions (such as execution time or memory utilization), applies Pareto optimization and, as a result, prepares a vector with information on the application execution parameters. The vector with identified application execution parameters is then returned to the scheduler. Based on the execution parameters, the scheduler prepares a set of possible application schedules to resources and deploys the application across the exascale infrastructure. During the execution, the whole process is monitored by the ASPIDE monitoring system, which then provides the gathered information to the ASPIDE EDE. The ASPIDE EDE examines if the possible execution parameters induced communication bottlenecks or irregular application behavior (including high execution time or memory utilization). This information is provided to the autotuner through a time series DB for future reference. Based on the information from the EDE, the autotuner can remove unsuitable execution parameters for future executions. If none of the parameters, identified by the autotuner, meet the execution requirements identified by the EDE, the whole process is repeated from the beginning.

## 6 Application Case Study

To evaluate the performance and the behavior of the ASPIDE autotuner and events and anomalies detection engine, we selected a representative case study concerning the processing of social media information. The widespread use of social media platforms allows scientists to collect a huge amount of data posted by people interested in a given topic or event. This data can be analyzed to infer patterns and trends about people’s behaviors related to a topic or an event on a vast scale. Social media posts are often tagged with geographical coordinates and/or other information that allows applications to identify user positions, enabling mobility pattern analysis using trajectory mining techniques. This case study aims to discover frequent trajectories from people’s movements to find the common routes followed by social media users. These most common trajectories occur between places-of-interest (PoI) that users visit in an area. A Region-of-Interest (RoI) represents the geographical boundaries of the PoI’s area (Belcastro et al., 2018). Still, it can also be defined as “a spatial extent in geographical space where at least several user trajectories pass through” (Giannotti et al., 2007).

The goal of the application is to process geotagged social media items by exploiting the metadata they contain to extract user trajectories. The input of our method is a large set of geotagged items gathered from Flickr. We used about 2.3 million geotagged items published in Flickr from January 2006 to May 2020 in Rome (named *dataset-Rome*). A geotagged item is a JSON string that contains a set of metadata elements. For example, a Flickr item includes the following elements: photo id, information about the user who published the photo, title, and description, the date on which the photo was taken, the format of the photo, the location where the photo was taken, and tags that describe the photo. To show the basic characteristics of data, an example of a Flickr post is shown in Listing 1.

Listing 1 Metadata of a Flickr postserialized in JSON format.

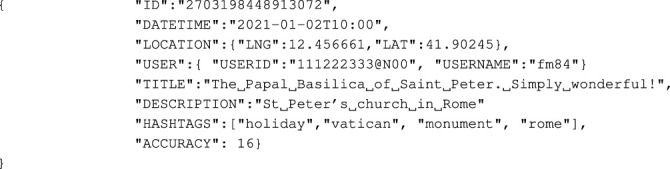

**FIGURE 5:**
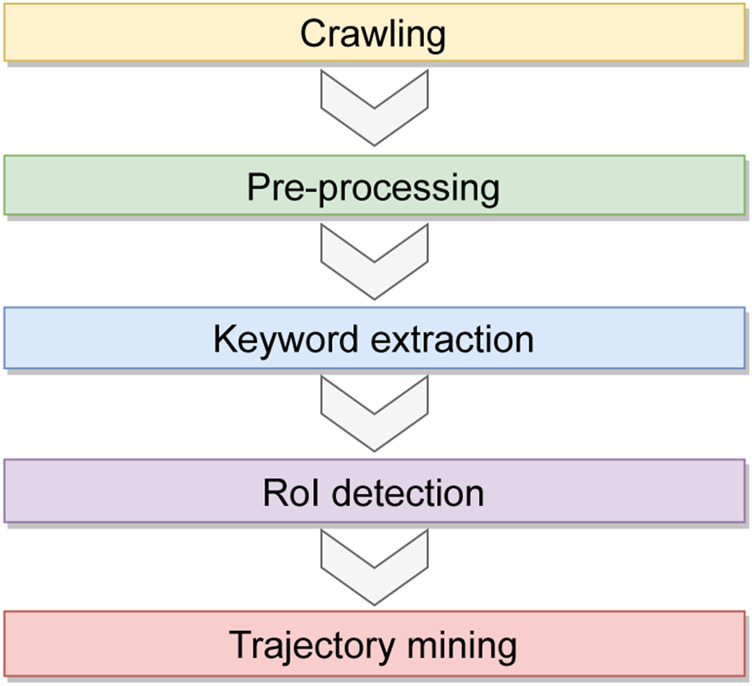
Workflow of the urban computing use case application.

The workflow is composed of the five main tasks, depicted in Figure 5:

A. Crawling: large amounts of data are collected from Flickr using public APIs or loaded from files stored on distributed file systems. Preprocessing: Flickr posts are preprocessed to make them suitable for analysis.
B. For example, posts can be filtered out if they do not meet some conditions.
C. Automatic keywords extraction and data grouping: the keywords that identify the places-of-interests are extracted; these keywords will be used to group social media items according to the places they refer to.
D. RoIs detection using a parallel clustering approach: A data-parallel approach is used to detect Regions-of-Interest (RoIs) starting from social media data grouped by keywords (Belcastro et al., 2020). RoIs represent a way to partition the space into meaningful areas; they are the boundaries of Points-of-Interest (e.g., a city square).
E. Trajectory mining: This step is run to discover the behavior and mobility patterns of people by analyzing geotagged social media items (Belcastro et al., 2019). To implement this step, the sequential pattern mining algorithm Prefix-Span (Han et al., 2004) is used.

The workflow has been implemented in Apache Spark. The main parameters used to configure the execution of the application are as follows:

• –dataset-path: path to the input dataset. To test the application with increasing datasets, starting from the initial dataset with a size of 108 MB, we created the 2X (219 MB), 4X (439 MB), 6X (658 MB), and 8X (880 MB) datasets.
• –threads-count (-t): number of threads.
• –number-of-partitions (-n): number of partitions used for data frame.
• –executor-memory (-e): the amount of memory reserved for Spark executor.

It is important to specify that our workflow can analyze items collected from Flickr and from other social media platforms, as done in some previous works with Twitter (Cesario et al., 2015) and Instagram (Cesario et al., 2017). In fact, the workflow requires: $i\left.\right)$ a collection of geotagged items; $ii\left.\right)$ such items must contain metadata with textual information (e.g., title, tags, and description); and $iii\left.\right)$ each user must be associated with different items so as to be able to extract a trajectory for each user and thus find the most frequent trajectories of a set of users.

## 7 Experimental Evaluation and Discussion

### 7.1 Autotuner

#### 7.1.1 Evaluation Testbed

To expose the versatility of the autotuner and evaluate its performance, we select a parallel system containing Intel Core i7-5500 with 8 GB RAM per node. Each CPU provides two cores and four threads, each with a base frequency of 2.4 GHz and a turbo boost frequency of 3.0 GHz.

For the experiments, we use the urban computing case study described in Section 6 with the initial dataset with a size of 108 MB. To execute the application case study, we use Docker and Ubuntu 20.04. To analyze the behavior with different numbers of CPUs, we limit the number of available CPU resources a container can use with the -cpus flag of Docker. We conduct experiments with 1, 2, and 4 CPUs.

For each experiment, we run the autotuner 50 times resulting in 50 application parameter solutions per experiment. As presented in Table 1, we used the following three application parameters: threads count (-t), number of partitions (-n), and executor memory (-e).

**TABLE 1:** Use case application parameter definitions.

Flag	Description	Definition range
-t	Number of threads for Spark driver in local execution	(1, 16)
-n	Number of partitions used for Dataframe	(1, 600)
-e	Amount of memory reserved for Spark executor	(50, 8000) MB

#### 7.1.2 Pluggable Optimization Objectives

As a pluggable objective, for evaluation purposes, we utilize the execution time *E*_*a*_ and the memory footprint *M*_*t*_ of the case study application. We define the execution time of the application by measuring the execution time *T*_*t*_(*t*_*i*_, *c*_*l*_, *e*) of a given task *t*_*i*_ on a CPU *c*_*l*_ with a different number of execution threads *e* as the maximal execution time of all predecessors:

$$T_{t}\left(t_{i},c_{l},e\right)=\left\{\begin{array}{cc} p_{t}\left(t_{1},c_{l},e\right),\; & pred\left(t_{i}\right)=\emptyset ;\\ max\left(T_{t}\left(pred\left(t_{i}\right),c_{l},e\right)\right)+D_{t}\left(t_{i}\right)\; & pred\left(t_{i}\right)\neq \emptyset , \end{array}\right.$$ (1)

where $p_{t}\left(t_{i},c_{l}\right)$ is the processing time of task *t*_*i*_, *pred*(*t*_*i*_) is the processing time of the predecessors of task *t*_*i*_ as measured by the monitoring system, and *D*_*t*_(*t*_*i*_) is the time required for transferring the data to the task.

Therefore, the application execution time *E*_*a*_ is equal to the execution time *T*_*t*_ of the last task *t*_*k*_:

$$E_{a}=T_{t}\left(t_{k},c_{l},e\right).$$ (2)

Related to the memory optimization we consider the memory footprint *M*_*t*_ for processing task *t*_*i*_ with input data set *C*_*s*_ as follows:

$$M_{t}\left(t_{i},C_{s}\right)=\frac{C_{s}}{C_{m}\left(c_{l}\right)},$$ (3)

where *C*_*m*_(*c*_*l*_) is the available memory to CPU *c*_*l*_. This objective defines how big a fraction of the memory reserved for the application is currently used considering the size of the input data set *C*_*s*_.

#### 7.1.3 Evaluation Results

**FIGURE 6:**
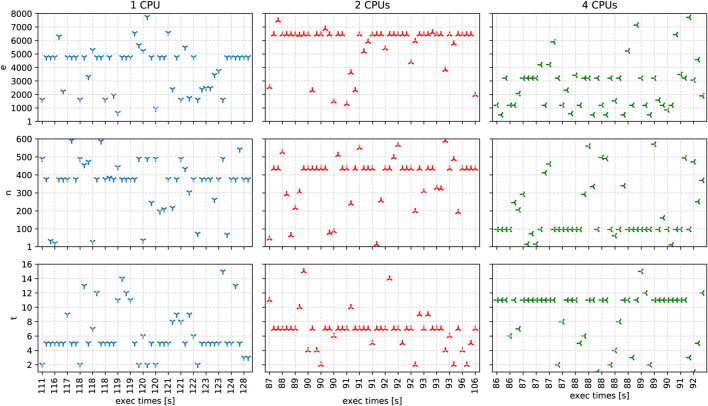
The solutions and their execution times in seconds, generated by the autotuner for one, two, and four CPUs after 50 iterations.

Figure 6 depicts our experimental results with one, two, and four CPUs, encompassing execution with the 8X (880 MB) dataset. Each solution has a separate *y*-axis corresponding to the three application parameters e, n, and t. To simplify the visualization of the solutions, we depict the number of available CPUs in correlation with the execution time as the *x*-axis. We display the different solution’s execution times with separate colors.

For one CPU, we observe that the fastest solution found has an execution time of 111s with 2 threads, 490 partitions, and 1612 MB of memory. Overall, the average values for threads are 6.22 ± 3.31, for partitions 353.56 ± 142.54, and for memory 4009.14 ± 1645.31.

For two CPUs, the fastest solution has an execution time of 87s with 11 threads, 46 partitions, and 2554 MB of memory. Four solutions achieve the second-fastest execution times of 88s with seven threads, a memory of at least 6449MB, and several partitions of 292, 526, and 434. Additionally, we also observe the slowest solution with an execution time of 106s close to the area of second-fastest solutions with 7 threads, 434 partitions, and memory of 1958 MB. Among all solutions, the average values for the threads are 6.77 ± 2.51, for partitions 375.36 ± 138.83, and for memory 5697.31 ± 1565.43.

For four CPUs, the autotuner determined the six fastest solutions, all with an execution time of 86s. The fastest solutions have different combinations of the following application parameter values: for t (487, 1191, 2060, 3210), for n (97, 205, 246), and for e (6, 7, 11). Considering all generated solutions, the average values for threads are 9 ± 3.58, for partitions 190.48 ± 160.58, and for memory 2638.31 ± 1783.81.

In summary, our experimental results show that the ASPIDE autotuning algorithm proposes fast solutions for different CPU specifications. We observe some intuitive solutions, such as 2 threads with 1 CPU, but also unexpected solutions, such as 11 threads with 2 CPUs. As we observe some fastest and slowest solutions with small euclidean distance side by side in the search space, we conclude that the fine-tuning of application parameters for our case study application is highly sensitive for even small parameter value changes and can lead to improvements in the execution time by up to 20*%*, compared to the standard runtime parameters.

### 7.2 Events and Anomalies Detection Engine

#### 7.2.1 Evaluation Testbed

For the evaluation testbed, we have deployed both the ASPIDE monitoring solution and the case study application. We set up both platforms on two identical workstations. They run *Intel(R) Xeon(R) CPU E5-2630* with 12 CPU Cores, 32 GB ECC RAM, 2 TB SSD with a base clock speed of 2.30 Ghz. These workstations also contain an NVIDIA Tesla K20c GPU. The operating system installed on these machines is *Ubuntu 20.04*. GPU acceleration was only used in the case of Deep Learning models. However, all preprocessing steps are executed on the CPU (such as data formatting and augmentation). Theoretically, it is possible to accelerate some preprocessing steps, but in the case of the experiments detailed in this article, the datasets easily fit into the workstations working memory, thus minimizing memory reallocation overhead.

We used an anomaly inducer solution to induce four types of anomalies, as described in the Section 4.3. It is important to note that these experiments were performed in such a manner to validate the EDE functionalities and integration with the ASPIDE framework.

**FIGURE 7:**
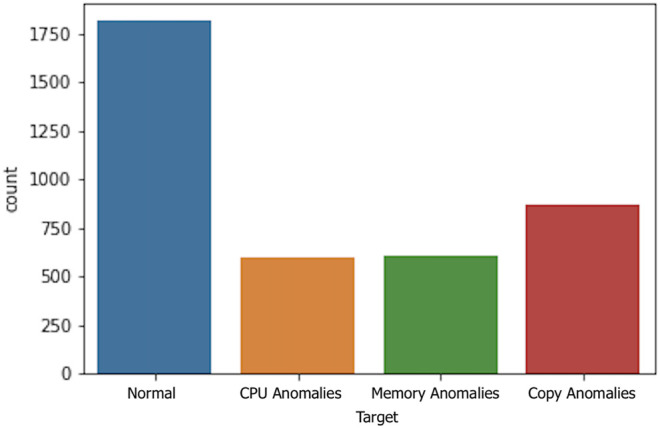
Distribution of event/anomaly classes.

Figure 7 shows the distribution of anomalous instances induced by the anomaly inducer tool. We can see that most events (marked with 0) are normal events and the rest of the anomaly occurrences have a similar distribution. It is important to note that true anomalies will most likely occur at a far lesser degree in a real-world scenario. In this case, we wanted to gauge the ability of each predictive model to detect anomalies without dealing with extremely imbalanced data sets.

We run the case study application on the largest data set (8x with a compressed file size of 880 MB). We limited the execution time of the application to 1 h. We set up the ASPIDE monitoring to collect metrics every second, resulting in 3900 data points containing 67 features. After the execution of the preprocessing steps, we were left with 63 features. No further preprocessing and feature engineering was done during these experiments.

#### 7.2.2 Evaluation Results

##### 7.2.2.1 Hyperparameter Optimization

The first stage of the experiments encompasses evaluating the hyperparameter optimization (HPO) technique with RandomForest, XGBoost, and DNN. This allowed us to optimize the performance of each predictive model separately. Each HPO iteration was executed until the model reached 1000 epochs. Table 2 shows the genetic algorithm parameters used during optimization. Due to technical limitations, it was necessary to use a distinct parameter space in the DNN models. This was largely because, unlike the other two methods, the DNN training can only be executed sequentially as it is reliant on a GPGPU. Furthermore, all inputs had to be hot encoded; thus, the type of cross-validation score used had to support this type of ground truth (*StratifiedKFold* does not support one-hot encoding).

**TABLE 2:** Genetic Algorithm parameters.

ID	Scorer	Splits	CV type	Jobs	Population	Tournament size	Mutation prob.	Crossover prob.
N	Accuracy	*4*	*StratifiedKfold*	*-1*	*40*	4	0.2	0.5
D	Accuracy	*2*	*Kfold*	*1*	*20*	4	0.2	0.5

**FIGURE 8:**
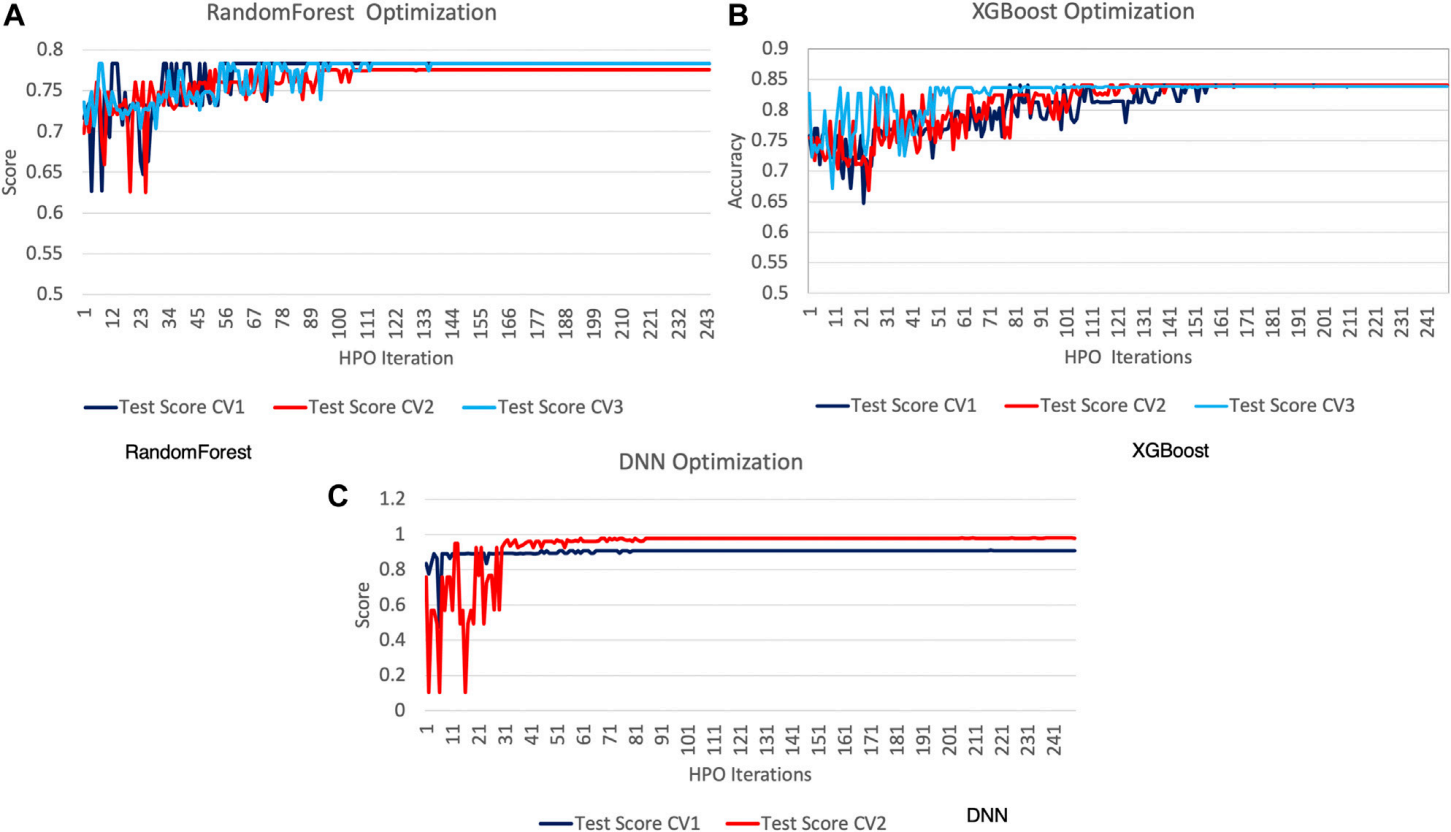
Hyperparameter optimization experimental results.

Figure 8 shows the HPO algorithm score for each of the optimization runs and the three cross-validation scores (CV1, CV2, and CV3). We can observe that RandomForest and XGBoost have considerable score churn, while DNN has a greater amplitude with a shorter churn duration. The DNN optimization score converges in our experiments 70 to 120 generations before the other methods, which proves its suitability for detecting anomalies. Another distinction evident from Figure 8 is that only two experiments were executed in the case of DNN. Although we used early stopping during training for each phenotype, the total training times of the DNN were significantly longer compared to the other two methods. Therefore, the execution of the DNN was limited to two experiments, as longer execution times could hinder the performance of the autotuner, which relies on the timely detection of the events and anomalies.

##### 7.2.2.2 Accuracy Evaluation

**FIGURE 9:**
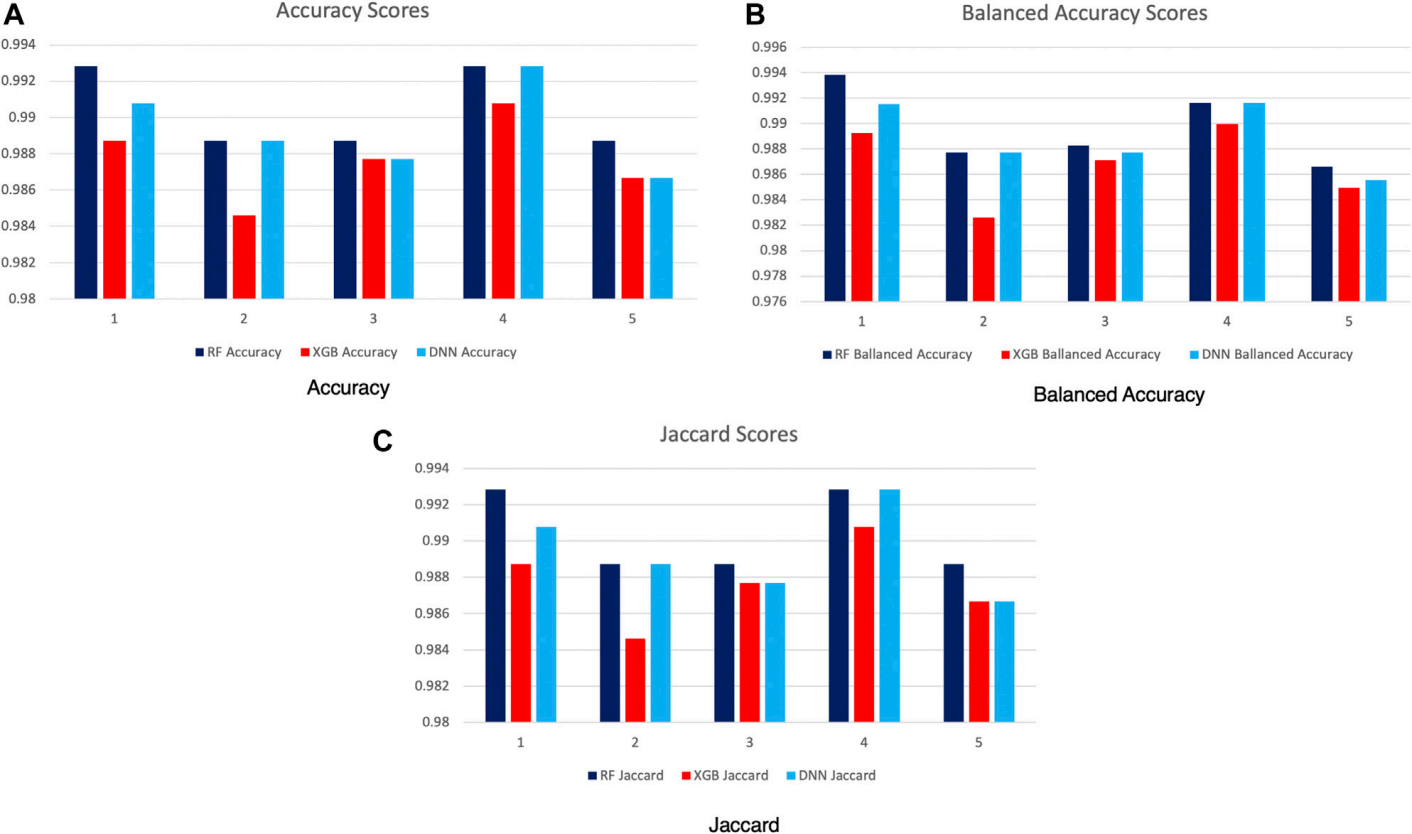
Model scores with cross-validation (5-fold).

In Table 3, we can see the parameters used for the best performing phenotypes from each of the three methods. Considering that the scores presented in Figure 8 are largely pertinent to the optimization stage, we needed to individually evaluate the best performing parameter space solutions. To this end, we run the second stage of our experiments on the highest scoring parameters for each method using 5-fold cross-validation while measuring the model accuracy, balanced accuracy (García et al., 2009), and the Jaccard index (Hamers et al., 1989). We can see the resulting scores in Figure 9. We can observe that all models performed well. The difference between the lowest and highest obtained accuracy is less than 0.02. Fold number 4 is of particular interest as it provides the highest average score between all three scoring functions for all predictive models.

**TABLE 3:** Best performing parameters.

RandomForest		DNN		XGBoost	
Parameters	Values	Parameters	Values	Parameters	Values
*n_estimators*	10	*optimizer*	Adam	*n_estimators*	1000
*max_depth*	50	*learning_r*	0.01	*max_depth*	4
*max_features*	50	*kernel_init*	he_normal	*learning_rate*	0.01
*min_samples_split*	5	*layer_0*	50	*sub_samples*	0.2
*min_samples_leaf*	6	*layer_1*	0	*min_child_weight*	6
		*layer_2*	50	*gamma*	0
		*layer_3*	100		
		*Drop*	0.3		
		*activation_1*	Relu		
		*out_activation*	Sigmoid		

**FIGURE 10:**
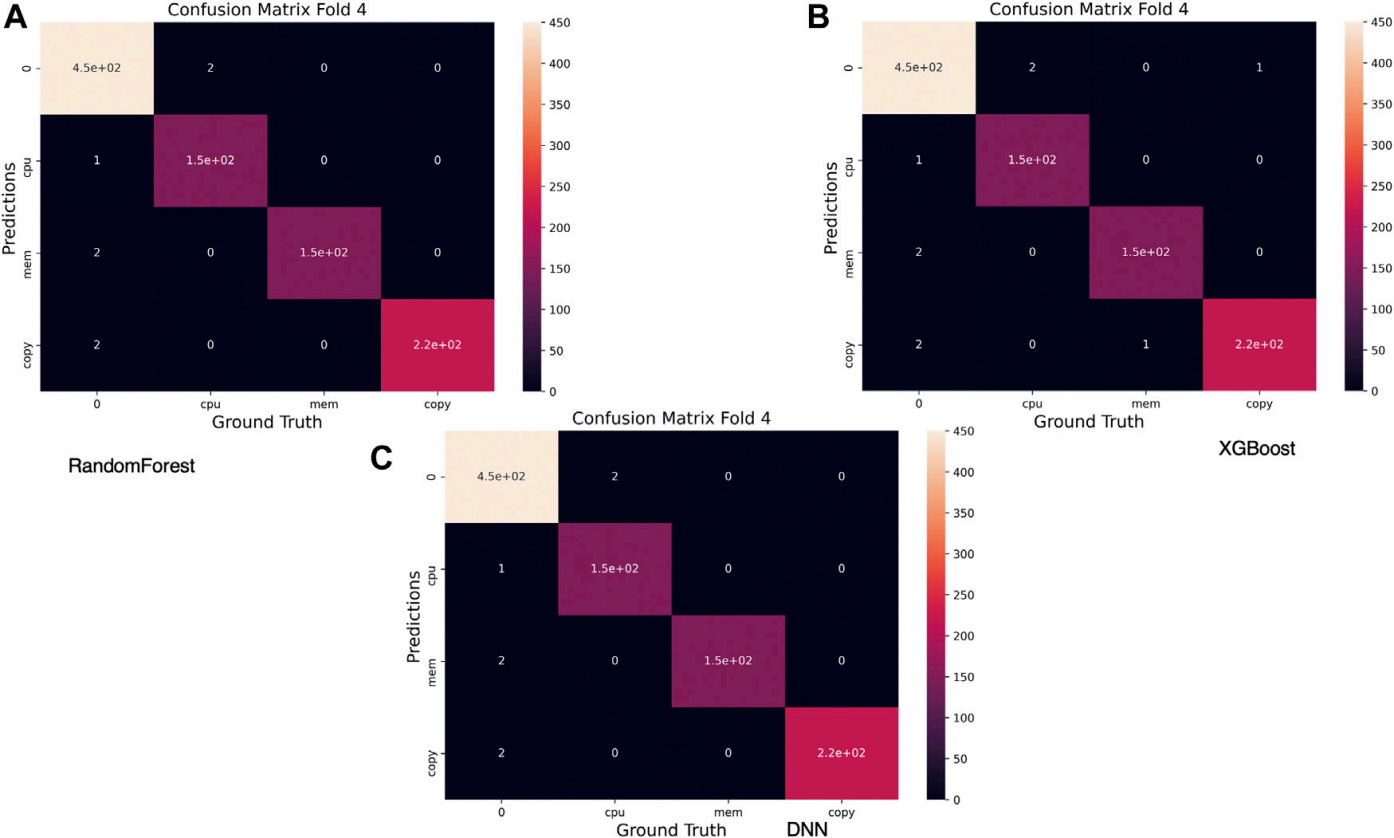
Confusion matrix for best performing validation fold.

Figure 10 depicts a heatmap representation of the confusion matrices for all three predictive models. The misclassification rate for all models is meager as most of these instances are related to classifying non-events as anomalies or anomalies as non-events. One exception is that in the case of XGBoost, where an anomaly was identified incorrectly as (*copy* instead of *mem*),

Finally, we provide a classification report, shown in Table 4, for the best performing of the three explored methods, namely, the DNN model. The resulting predictive model, using the identified parameters, has performed very well in terms of accuracy. However, one should not consider the validated model and the used parameters as optimum in all circumstances. There are no true minority classes in the validation dataset, and there are a substantial number of events representing each class, limiting the application of the best performing DNN model.

**TABLE 4:** DNN classification report fold 4

	Precision	Recall	f1-score	Support
Normal	0.9891	1.0000	0.9912	455
CPU anomalies	0.9903	0.9933	0.9908	150
Memory anomalies	1.0000	0.9901	0.9934	152
Copy anomalies	1.0000	0.9908	1.0000	218
accuracy			0.9918	975
macro avg	0.9923	0.9911	0.9917	975
weighted avg	0.9919	0.9918	0.9918	975

## 8 Conclusion

In this article, we introduced an exascale autotuning approach based on an NSGA-II multi-objective optimization algorithm integrated within the ASPIDE exascale computing framework. The approach considers multi-dimensional search space to support the utilization of pluggable objective functions, including execution time and memory utilization. Furthermore, the autotuner employs a machine learning-based event detection approach to detect anomalies during execution, such as hardware failures or communication bottlenecks. We utilize the events and anomalies detection engine to constrain the search space of the optimization problem, thus further improving the execution efficiency of the exascale applications.

We evaluated the ASPIDE autotuner on a representative social media application and corresponding data set. We have created experimental scenarios in both simulation and a real-world testbed. Our results show that the ASPIDE autotuner can reduce the execution time of exascale applications by up to 20% while maintaining a low memory utilization ratio of 2638 MB. Furthermore, the event detection engine achieved an average accuracy of 95% for detecting CPU- and memory-related errors, which significantly increased the execution efficiency of the case study application.

In the future, we plan to extend the autotuning model to support application autotuning for GPUs and conduct experimentation with more case studies over larger exascale infrastructures.

## Data Availability

The data is available at the EU commission platform Zenodo, https://zenodo.org/.

## Footnotes

1
https://www.aspide-project.eu/

2
https://www.tensorflow.org/

3
https://github.com/DEAP/deap
